# Alternative financing mechanisms for ART programs in health facilities in Uganda: a mixed-methods approach

**DOI:** 10.1186/s12913-017-2009-6

**Published:** 2017-01-23

**Authors:** Henry Zakumumpa, Sara Bennett, Freddie Ssengooba

**Affiliations:** 1 Makerere University, School of Public Health, Kampala, Uganda; 2 Johns Hopkins University, Bloomberg School of Public Health, Baltimore, USA

**Keywords:** Sustainability, ART scale-up, HIV funding, Health systems, Implementation research, PEPFAR, Mixed-methods, ART scale-up, Global health initiatives

## Abstract

**Background:**

Sub-Saharan Africa is heavily dependent on global health initiatives (GHIs) for funding antiretroviral therapy (ART) scale-up. There are indications that global investments for ART scale-up are flattening. It is unclear what new funding channels can bridge the funding gap for ART service delivery. Many previous studies have focused on domestic government spending and international funding especially from GHIs. The objective of this study was to identify the funding strategies adopted by health facilities in Uganda to sustain ART programs between 2004 and 2014 and to explore variations in financing mechanisms by ownership of health facility.

**Methods:**

A mixed-methods approach was employed. A survey of health facilities (*N* = 195) across Uganda which commenced ART delivery between 2004 and 2009 was conducted. Six health facilities were purposively selected for in-depth examination. Semi-structured interviews (*N* = 18) were conducted with ART Clinic managers (three from each of the six health facilities). Statistical analyses were performed in STATA (Version 12.0) and qualitative data were analyzed by coding and thematic analysis.

**Results:**

Multiple funding sources for ART programs were common with 140 (72%) of the health facilities indicating at least two concurrent grants supporting ART service delivery between 2009 and 2014. Private philanthropic aid emerged as an important source of supplemental funding for ART service delivery. ART financing strategies were differentiated by ownership of health facility. Private not-for-profit providers were more externally-focused (multiple grants, philanthropic aid). For-profit providers were more client-oriented (fee-for-service, insurance schemes). Public facilities sought additional funding streams not dissimilar to other health facility ownership-types.

**Conclusion:**

Over the 10-year study period, health facilities in Uganda diversified funding sources for ART service delivery. The identified alternative funding mechanisms could reduce dependence on GHI funding and increase local ownership of HIV programs. Further research evaluating the potential contribution of the identified alternative financing mechanisms in bridging the global HIV funding gap is recommended.

## Background

In the past decade, the dramatic expansion in antiretroviral therapy (ART) coverage in Sub-Saharan Africa (SSA) depended substantially on Global Health Initiatives (GHIs) notably The Global Fund to Fight AIDS, Tuberculosis and Malaria (GFATM) established in 2002 and The President’s Emergency Fund for AIDS Relief (PEPFAR) commissioned in 2003 [1, 2].

After years of sustained growth in international funding for the global HIV response, recent indications suggest that international funding has plateaued [3, 4]. In 2015, although international funding remained steady at $ 10.8 billion, compared to 2013, $ 11.2 billion was raised indicating a 3.3% decline [5].

Against this backdrop of uncertainty in the long-term sustainability of international funding for ART service delivery in SSA and shifting donor priorities, there are amounting calls for alternative funding streams [6, 7]. The 2011 UN Political Declaration on HIV and AIDS called for ‘accelerating efforts to identify innovative funding mechanisms’ beyond the traditional funding sources to sustain and further expand ART coverage [8]. Whereas several countries in Sub-Saharan Africa increased domestic investment in their national HIV response between 2006 and 2011 [9], a substantial funding gap still remains [10]. There are renewed calls for locally-led alternative funding streams for bridging the resource gap for meeting ART scale-up targets in Sub-Saharan Africa [4, 6, 8].

Uganda has a generalized HIV epidemic with an estimated 1.7 million people living with HIV [10]. A national emergency ART scale-up program was implemented between 2004 and 2009 with external donor support. ART services were initially piloted at the national and regional referral hospitals with a gradual scale-up to lower level public health facilities [11, 12]. Under the USAID-funded Health Initiatives for the Private Sector (HIPS) project, for-profit health facilities were supported to start ART services [13]. Private not-for-profit (PNFP) health facilities were supported under multiple PEPFAR implementing partners and Global Fund [11, 12].

The population in need of ART in Uganda continues to increase. In 2012, The National AIDS Indicator survey revealed that national HIV prevalence rates had increased from 6.4% in 2005 to 7.2% in 2011 [14]. In 2013, Uganda together with South Africa and Nigeria accounted for almost half of all new HIV infections in SSA [15]. The number of Ugandans enrolled on ART in 2015 was 763,720 which represents about 46% of the population living with HIV [10]. WHO ART treatment guidelines of November 2015 require that all diagnosed with HIV be enrolled on ART regardless of CD4 count [11]. Uganda committed to enrolling 80% of those with HIV on ART by 2020 in the National HIV and AIDS Strategic Plan. Furthermore, patients enrolled on ART are living longer, compounding total estimates of future need. The mounting fiscal pressures to treat the accumulating number in need of ART renders alternative financing channels critical in Uganda.

### Current funding sources for HIV service delivery in Uganda

Uganda is heavily dependent on external donors with over 85% of the national HIV response funded through bilateral and multilateral partners [4]. PEPFAR and The Global Fund are the largest funding sources for the national HIV response [16]. The Global Fund provides the majority of funding for ART commodities in public facilities which constitute about 62% of all ART sites in Uganda [17]. An estimated 95% of all Global Fund grants go to the procurement of commodities through a centralized public medicines supply system. PEPFAR funds most of the ART commodities for private for-profit and private not- for-profit health facilities (including those housed in government hospitals) [16]. Many of the HIV prevention programs in Uganda such as prevention of mother to child transmission (PMTCT), Safe Male Medical Circumcision and several other HIV prevention efforts are supported by PEPFAR through multiple national and international ‘implementing partners’ [4, 16]. Additionally, PEPFAR provides funding that supports ART services delivery such as on-site support supervision to ART service providers, workforce training and program reporting support [11, 16].

The Uganda government contributes about 15% of the costs of the national HIV response [10]. In 2013, 24% of the cost of procuring antiretroviral drugs (ARVs) was met through national budget support [16]. Furthermore, the government indirectly supports ART service delivery through health systems budget support such as through salaries for health workers in the public sector. Of the 7.4% of the government budget devoted to the health, only about 3% goes to the HIV response [4].

There have been several studies examining the financial sustainability of ART programs in SSA from a dimension of global investments for ART scale-up [18–20]. On the other hand, studies have taken the perspective of increasing domestic government spending [21, 22]. However, few studies have explored the financial sustainability of ART programs at the organizational-level [23].

Many previous studies have adopted quantitative approaches using unit costs analyses to model HIV treatment needs [24–26], but there has been limited in-depth investigation into health facility contexts and the strategies adopted at the organizational-level to sustain ART programs. Moreover, the perspectives of front-line ART program managers in Sub Saharan Africa on the financial sustainability of ART programs remain under-explored given the dominant top-down discourses. The objective of this study was to identify the funding strategies adopted by health facilities in Uganda to sustain ART programs between 2004 and 2014 and to explore variations in financing mechanisms by ownership of health facility.

We situate this study within the analytical framework by Shediac-Rizkallah & Bone [27] in as far as it addresses the sustainability of ART interventions within implementing organizations in Uganda.

This study is derived from a doctoral research project investigating the sustainability of ART programs in health facilities in Uganda from the perspective of the interactions in the six building blocks of the health system [11, 17, 28].

## Methods

### Study design

A mixed-methods research design was employed involving quantitative and qualitative data collection and analysis [29]. The study was conducted in two phases applied sequentially [30].

### Study population

The study population comprised of health facilities in Uganda which were accredited to provide ART between 2004 and 2009. Health facilities were categorized by ownership; i) Public (ii) Private for-profit (PFP) and iii), Private not-for-profit (PNFP) [31]. Participating health facilities were drawn from the different levels of care of the Ugandan health system. This ranged from the tertiary level (National and sub-national referral hospitals), secondary level (District hospitals) and primary level (county and sub-county health centres and clinics) [12, 32].

### Sample selection

#### Quantitative phase

Firstly, we obtained the published Ministry of Health ART Unit Monitoring Report of March 2010 which lists 394 accredited ART Clinics in Uganda, as at the end of 2009. The list contained details of the districts where the clinics were located and their ART program characteristics such as patient loads. Secondly, we selected participating health facilities based on Uganda’s 10 geographic sub-regions [33]. All 394 ART clinics were grouped into ten clusters. We randomly sampled health facilities from the 10 clusters based on proportionate representation retaining a sample of 195 health facilities.

#### Qualitative phase

From the national sample of 195 hospitals, six health facilities were purposively selected. Health facilities were selected to represent the three ownership categories; Public, PFP and PNFP. Table 1 shows that two health facilities were selected from each of the three ownership categories.

**Table 1:** Description of health facilities selected for in-depth study

Ownership category	Health facility	Setting	Level of care	Year ART commenced	Number of ART grants (2009–2014)
Public	P-001	PERI-URBAN	Hospital	2004	3
	P-002	URBAN	Health Centre four	2006	2
Private not for profit	PNFP-001	URBAN	Hospital	2005	5
	PNFP-002	RURAL	Health Centre four	2005	5
Private for profit	PFP-001	URBAN	Hospital	2006	1
	PFP-002	URBAN	Health Centre four	2009	1

We aimed for an appropriate rural/urban mix and ensured that at least half of the health facilities were based in rural areas. We struck a balance between health facilities which had well-established ART programs with those which had less-established ART programs (based on year ART was first implemented, staffing strength and patient volumes).

### Data collection

#### Quantitative

The head of the ART clinic in each of the health facilities (*N* = 195) filled a pre-tested questionnaire comprising both closed-ended and open-ended questions. The closed-ended questions aimed at generating data relating to the sources of funding for ART service delivery, the nature of support received from funders and the number of ART funders in the last 6 years. The open-ended questions inquired into the funding strategies adopted by health facilities to sustain ART service delivery since the national scale-up program. The questionnaire was divided into four sections. The first section contained questions regarding provider characteristics such as the ownership category, level of care and range of HIV services offered. The second section aimed at generating data relating to ART funding sources, alternative financing strategies devised by providers. The third section was concerned with assessing projected ART program sustainability. Data were collected between January and April 2014.

#### Qualitative

An interview guide was constructed based on findings from the quantitative phase. Semi-structured interviews (*N* = 18) were conducted with three respondents from each of the six health facilities (leadership, finance managers, ART Clinic management). Interviews lasted between 45 and 60 min and were audio-taped by the first author. The interviews were typically conducted in interviewees’ offices. Interviews were conducted between February and June 2015.

### Data analysis

#### Quantitative

Data from the questionnaires were edited, cleaned and initially entered into Epi Data (version 3.1) software. Data were then exported into STATA (version 12) where descriptive statistics were generated to describe the characteristics of the participating health facilities. Frequency counts and percentages relating to the sources of finance and funding strategies for ART programs at the surveyed health facilities were analysed.

Statistical tests of association were conducted where potential independent variables (ownership of health facility, number of ART funders) and the outcome variable which was operationalized as a question. *How confident are you that ART services will still be active at your health facility in 5 years’ time?* This was measured on a 3-point Likert Score (1. Not at all 2. Somewhat 3. Very) [34]. Statistical analyses were performed using chi-square tests with the level of statistical significance set at 95% confidence interval (*p* = 0.05).

#### Qualitative

The semi-structured interviews with ART clinic managers were transcribed verbatim by two authors. The authors separately read the interview transcripts multiple times for data familiarization and inductively devised an initial coding scheme. Subsequently, the authors compared coding schemes and merged them into one through a team-based consensus. A visual representation of our qualitative data analysis procedures are shown in Table 2.

**Table 2:** Summary of qualitative data analysis procedures

	Data collection period	Processes	Outputs
*Phase One*			
Mixed-methods survey *(n = 195)*	January- April 2014	Analysis of open-ended text relating to funding strategies adopted	Codes of funding strategies adopted by health facilities
*Phase Two*			
In-depth study *(n = 6)*	February- June 2015	Constructing Interview guide based on emergent codes from survey	Interview protocol
		Conducting face-to-face semi-structured interviews with ART Clinic managers *(n = 18)*	Audio recordings of interviews
		Transcribing interviews into text data	Interview transcripts (*n* = 18)
		Coding of interview transcripts	Codes generated
		Collapsing codes into themes	Themes developed

## Results

### Characteristics of the sample

A total of 195 health facilities participated in the study. In terms ownership of health facility, 121 were public facilities, 35 were private not-for-profit (PNFP), 33 were private for-profit (PFP) and 6 were HIV Research Clinics.

With regard to setting, 88 (45%) of the health facilities were based in peri- urban areas, 76 (39%) were in urban areas and 27 (14%) that were in rural areas. Table 3 shows that the 195 health facilities were based in 38 districts of Uganda drawn from all of Uganda’s ten geographic sub-regions as designated by The Uganda Bureau of Statistics [33].

With regard to level of care, Health Centre IVs were the most represented 72 (37%), followed by hospitals 58 (30%), clinics at 33 (17%) and Regional Referral Hospitals at 12 (9.3%).

Of the 195 respondents, 52.8% were male (*N* = 102) and 47.2% were female (*N* = 92). The most represented cadre of health workers who participated in the survey were Clinical Officers 66 (33.9%) followed by nurses 58 (30%) and medical doctors 43 (22.5%).

## Sources of funding for ART service delivery

The majority of participating health facilities 183 (94%) reported a PEPFAR implementing organization as a source of funding for ART service delivery in the last 6 years preceding data collection in April 2014. The Global Fund was cited as a funding source by 7 (4%) of the health facilities. AIDS Health Care Foundation (AHF), a private US-based philanthropic organization, was reported as the principal funding partner for ART service delivery in five health facilities in South-Western Uganda.

In PEPFAR’s case, funding was reported to be channeled through intermediary organizations known as ‘implementing partners’ under time-limited project cycles. It was observed that PEPFAR-implementing organization partners were assigned geographic zones since 2009/2010 which catered to ART- providing organizations within those zones.

### Multiple sources of funding

Of the 195 participating health facilities, 161 (72%) reported having at least two concurrent grants supporting ART service delivery between January 2009 and April 2014. There were variations in the number of donors reported by ownership of health facility. PNFPs reported a large number of donor inputs than any other health facility category. On average, public facilities reported two donors compared to private not-for-profit which reported 3.5 donors and private for-profit health facilities which had an average of 1 donor in the last 6 years preceding data collection in April 2014.

The qualitative evidence demonstrated that the support received from government and GHIs for ART service delivery at the participating health facilities was insufficient and only covered the core components of ART delivery such as ARV drugs and commodities. ART was described as a complex intervention with many arms and facets. The costs of treating HIV-associated opportunistic infections (OPIs) emerged as a grey area that was not supported under status-quo funding in the surveyed health facilities.

*Who pays for treating opportunistic infections? If the patient has a skin rash and they need an antibiotic of sorts, who pays for that? Our main funder doesn’t.* [IDI 1201]

*He will come with malaria and another day with diarrhea. You know they get sick often. But opportunistic infections (OPIs) are not covered under the ART package.* [IDI 1302]

ART providing organizations reported seeking supplemental funding from a variety of sources from the external environment for non-core components of ART delivery not supported by government or GHIs which have been defined as bilateral and multilateral financing mechanisms for disease control [35]. Private philanthropic organizations and individual donors emerged as important sources of supplemental funding particularly in private not-for-profit health facilities.

*There are areas which our main donor doesn’t support so we bring in other donors to support these grey areas. We have friends from abroad. The Good Will Ambassadors. Some are one-off grants. Someone has 5,000 US dollars and asks “ how do you want to use it?”* [IDI 1202].

Interviews with providers also revealed that health facilities sought multiple funding channels because of the time-limited nature of GHI funding and the volatility associated with donor grants. Providers variously recounted experiences of coping with discontinued funding arising from the ending of project grants but also in some cases, abrupt closure of ART service delivery grants.

*In 2009, our main project grant ended. We cut our staff in half and computerized as much of our operations as we could* [IDI 1103].

*In 2014, when Uganda got in the news about passing an anti-gay law, funding to our implementing partner was stopped. We were stuck with hundreds of patients here* [IDI 1202].

### The augmenting role of philanthropic aid

The nature of support sourced from philanthropic organizations and private individuals was diverse and ranged from infrastructure support such as ART clinic expansion to nutrition support for HIV patients. In one of the health facilities participating in the study, the Tides Foundation was supporting a model of integrating HIV with family planning services, overseas private individuals and the Triangle Community Foundation were funding livelihoods support of HIV patients and Janssen Global Public Health R&D provided funding for ART adherence and opportunistic infections treatment support. In a PNFP facility, the Harold Foster Foundation supported nutrition support for ART patients, Historic Christ Church donated medical equipment to support ART service delivery.

There was a statistical significance in the association between health facilities which reported more than one ART grant with level of confidence in with ART program continuation in 5 year’s time (*p*-value of 0.003).

### Different funders for different components of the ART program

An important finding of this study is that ART-providing organizations sought alternative funding sources for separate arms of their ART programs (Table 4).

**Table 3:** Participating districts by geographic sub-region for questionnaire survey

Geographic sub-region	Districts covered
Northern	Lira, Gulu, Pader, Kitgum
Central 1	Wakiso, Gomba, Masaka, Lwengo, Kalungu, Rakai, Bukomansimbi
Central 2	Luwero, Nakaseke, Mityana, Kayunga, Buikwe, Mukono
Western	Bundibugyo, Kasese, Kabarole, Kyegegwa, Kyenjojo, Kibaale, Hoima, Masindi
Kampala	Kampala
South Western	Mbarara, Kabale, Ntungamo
Eastern	Tororo, Mbale
West Nile	Moyo, Arua, Nebbi
East Central	Jinja, Iganga, Mayuge
Karamoja	Moroto

**Table 4:** ART program component and funding partner

Facility ownership category	ART intervention component	Funder
Health Facility A (PNFP)	Medicines for opportunistic infections	Catholic International Development Charity-UK
	Laboratory support	Goita-Ireland
	ARV drugs and testing kits	Ministry of Health Uganda
	Operating systems and systems strengthening	GOAC-International
	Training of ‘Expert patients’	McCarthy Foundation
Health Facility B (Public)	ARV drugs and workforce training in ART	Ministry of Health Uganda
	Laboratory support and client follow-up	Infectious Diseases Institute (IDI)
	Laboratory reagents, community outreaches	SUSTAIN-Uganda (USAID)
	Multivitamins and nutrition support	Private missionary
	Pediatric care and capacity building	Baylor College of Medicine

Having multiple donors for separate components of the same ART program was a trend that cut across all categories of facility ownership.

#### Projected sustainability of ART programs

In light of the reported levels of dependence on GHI funding for ART service delivery, we sought to assess projected likelihood of ART program continuation at participating health facilities. The head of the ART Clinic at each of the 195 health facilities was asked to indicate how confident they were that ART services would still be active in 5 years’ time [30]. The majority of health facilities 144 (74%) indicated that they were Very Confident. Thirty-five (18%) selected Somewhat Confident with only 8 (4%) choosing Not Confident. Varied reasons were elicited from providers in explaining their confidence in ART program continuation. They ranged from the perception that ART delivery processes had become integrated into routine organizational procedures over the last 12 years to those who framed it as a national and global health obligation on the part of funding partners.

*I think donors will continue to support HIV treatment in Uganda because it’s now an international moral obligation. Is it ethical to stop treatment for someone whom you started on these life-saving drugs and then stop midway?* [IDI 1211].

*Over the years government has built capacity and to carry on HIV service delivery even if donors pulled out. For example, ART commodities are supplied through National Medical Stores (the national medicines supplier) and most patients attend public health centres* [IDI 1116].

There was a significant association between level of confidence in ART program continuation and ownership of health facility (*p* < *0.001*). More PFPs selected Very Confident than any other ownership category. They were followed by public facilities with PNFP facilities showing the least confidence in ART program continuation in 5 years’ time.

## ART financing strategy by ownership of health facility

The open-ended responses in the questionnaire and statistical analyses suggest that ART program financing strategies differed by type of ownership of a health facility. This section presents results from the semi-structured interviews conducted with ART Clinic Managers and staff from the six selected health facilities representing Uganda’s three major health facility ownership categories.

## Private not for profit (PNFP)

The two PNFPs reported that they deliberately sought multiple donor grants as a strategy for sustaining ART interventions. Grant proposal writing was reported as an on-going process to secure new sources of financing from external funders. One of the PNFPs reported having a team of dedicated grant writers who were experienced in navigating the competitive donor funding environment. This was perceived to have been key in their ability to attract 5 ART service delivery grants in the last six years. Interviews with ART clinic managers from PNFPs revealed that they were more dependent on donor funding than other providers and writing multiple grant proposals to a wide range of funders was a strategic approach that enabled them to provide ART services without charging patients.

*We look for alternative sources of funding to keep the ART program afloat. We are constantly writing proposals to donors. We approach a variety of donors to keep the ART program running.* [IDI 1204].

*Over 70% of our HIV work depends on donor funding. Donor funding determines our patient enrollment capacity* [IDI 1203].

Compared to public facilities which depend on government subventions and private-for-profit health facilities which charge fees for the majority of their services, PNFPs don’t have assured funding streams and are therefore more donor-dependent for ART service delivery.

The qualitative interviews highlighted the role of external program champions who were described as individuals who were instrumental in securing resources for sustained ART provision by linking the health facilities with additional grant funding and in-kind resources from external sources. Some of the external champions were foreign nationals who had helped found the ART clinics in the two PNFPs but had continued to secure additional funding even after returning to their home countries.

A modest fee of 2500 Uganda shillings ($ 1) was charged for every client visit to the ART clinic at PNFP-001. This clinic indicated an accumulative patient load of over 7000. With the clinic running throughout the week, this user fee constituted an important alternative revenue stream to cover un-funded aspects of ART service delivery.

*Who gives you patient chairs for the clinic? Who buys snacks for our pediatric patients when they come in for their sessions? That fee covers those small items* [IDI 1201].

Adopting an entrepreneurial posture was a necessity for securing resources from funding sources in the external environment. One of the PNFPs indicated that being a center of excellence in HIV care and treatment in Uganda, they hosted a national HIV Clinicians training institute. Through this platform, they sought and attracted international grants and government contracts for regular trainings of ART clinicians from all over Uganda from which they leveraged operating costs for their wider ART program.

The introduction of a ‘VIP’ section of the ART clinic where a segment of high-end patients were charged fees for receiving an exclusive service offered after normal working hours was reported to be an additional income generating scheme in one facility. The revenue generated from VIP clinic services were said to support recurrent costs of the wider ART program.

## Private for- profit (PFPs)

Fee-for-service was the dominant funding stream in for-profit facilities. Participating PFPs indicated that with the exception of ARV drugs which were supplied without charge through a centralized national medicines supply system, patients were required to pay for other costs of HIV care and treatment. The services which were said to attract charges include treatment for opportunistic infections (OPIs) and laboratory investigations. Due to the integrated nature of service delivery in the participating for-profit providers, ART programs were reported to create demand for other sections of the health facilities such as the laboratory and pharmacy sections in a form of economic interdependence in revenue streams between ART and other health facility services.

### Charging lower consultation fees

To enhance the affordability of consultation fees by patients, for-profit health facilities reported charging a lower consultation fee for HIV patients compared to regular patients. In the PFPs studied, the consultation fee for HIV patients was several times lower than that of regular patients. A consultation fee of 10,000 Uganda shillings (US$ 4) was charged compared to the regular consultation fee of 50,000 Uganda shillings (US $20).

Reflecting the trend in PNFPs, for-profit health facilities also reported the introduction of Executive Clinics for patients who sought an exclusive service that afforded them privacy. These patients were reported to pay higher service fees compared to regular patients as a revenue-enhancement strategy for supporting the broader portfolio of patients.

The sale of brand ARV drugs to patients in PFPs who declined the publically-provided generic drugs was reported to boost revenue. The price of brand drugs to the patient was almost 10 times the cost of their generic versions and the proceeds generated helped offset some of the recurrent costs of the broader ART program at for-profit health facilities.

A special medical insurance scheme for HIV patients was introduced in 2010 at one of the facilities. For an annual premium of about 500,000 Uganda shillings ($ 250), HIV patients had their ART treatment costs covered under this umbrella scheme. The uptake of this scheme was reported to be uneven but had been intended as a mutually beneficial arrangement for enhancing patient affordability of costs associated with regular reviews and for the private clinic to deliver higher quality services at a lower cost. The items covered in the annual HIV medical insurance scheme are reflected in Table 5.

**Table 5:** Annual HIV medical insurance plan in PFP-002 in June 2010. Value of Insurance premium: Uganda shillings 500,000 ($250)

Items covered in insurance plan	
ARV drugs (*Not charged)*	Funded by PEPFAR through Joint Medical Stores and Medical Access
Laboratory diagnostic charges	Covered under scheme
Consultation fee per client visit	Covered under scheme
Drugs for treating OPIs	Covered under scheme
Medical consumables used during visit	Covered under scheme
Cotrimaxole prophylaxis	Covered under scheme

Apart from the special medical insurance scheme targeting their un-insured clients, the two PFPs reported that a number of their ART patients were covered under employee medical insurance schemes especially for those patients employed in the private sector which offered staff medical cover with private insurance companies.

## Public facilities

Interviews with ART clinic managers from the two selected public hospitals revealed that they sought additional funding for components of the ART program not supported from their principal sources of funding of government and GHIs. Several of the alternative funding streams cited were not dissimilar to those reported under private not- for-profit facilities. The additional funding was said to be sourced from private philanthropic organizations and individual donors with the role of external program champions highlighted as important in this respect.

Besides seeking external sources of funding, one of the two public hospitals also sought new funding opportunities available within the country. A period was cited when they had four separate funders supporting the ART program.

*In 2006, we had four different partners supporting our ART program. We had a western researcher mobilizing funds for multivitamins and nutrition support, a partner supporting our patient data base, drugs (ARVs) from government and a laboratory where we did viral load testing without charge* [IDI 1103].

A large public hospital reported leveraging funding from the multiple HIV research programs they hosted for local and international researchers to cover ART clinic overhead costs and salary top-ups for the workforce in their ART clinic.

## Discussion

We employ a mixed-methods approach to examine alternative financing mechanisms for ART programs in health facilities in Uganda which were accredited to provide ART services between 2004 and 2009. Our findings show that health facilities diversified funding streams as a strategy for the long-term sustainability of ART programs. In our national sample of health facilities, 72% reported at least two concurrent grants supporting ART service delivery in the last 6 years preceding data collection in April 2014. Private philanthropic aid and individual donors emerged as important sources of supplemental funding for ART delivery in participating health facilities. ART-providing organizations sought additional funding to address aspects of ART service delivery that were not supported by government and GHI funding partners. Our study found that ART program financing strategies differed by the ownership-type of a health facility. Despite heavy reliance on short-term donor grants for ART service delivery, the majority of health facilities (76%) were Very Confident of ART program continuation in the next 5 years.

Our finding that facilities sought to diversify funding sources is consistent with previous studies which show that having multiple funding streams enhances long-term program sustainability outcomes. LaPelle et al. [36] and Steven & Peikes [37] investigated factors associated with the sustainability of interventions and concluded that multiple funding streams heightened the likelihood of sustainability of interventions in implementing organizations. Recent systematic reviews have reported similar findings [38, 39].

### ART financing mechanisms by ownership of health facility

ART program funding strategies varied by the ownership-type of a health facility. Although some strategies such as multiple funding streams cut across all categories of health facilities, PNFPs reported writing multiple grant proposals to donors as a strategic funding strategy compared to other health facility ownership-types. We found that on average, PNFPs reported more donor grant inputs than any other health facility category. The finding that PNFPs reported more donor funding is well aligned with studies which suggest that non-governmental organizations are more successful in attracting international funding [40–42].

### Donor dependence

The time-limited grant cycles under PEPFAR leave ART providers and patients vulnerable to volatility due to shifting donor priorities and changing contracts. Larson et al. [43] call for long-term funding in HIV responses to realize impact in patient outcomes in place of the 1 to 2 year funding cycles that currently characterize HIV funding practices. Interviews with ART program managers suggest that sustained external funding of ART programs, over the 10-year study period, has coalesced into a culture of aid dependence. This has had the inadvertent effect of externalizing the challenge of sustained funding for the HIV response through diminishing the imperative for political and community ownership of HIV programs in donor-dependent countries. We observe that even when health facilities sought alternative funding streams for ART programs they were still predominantly from external sources. This could be partly attributable to Uganda’s development status and its classification as a low-income country. Shediac-Rizkallah and Bone [27] assert that short-term grants of 3 years or less impede sustainability and that ‘excessive outside funds (the supply-side) can inhibit sustainability’. On the other hand, Scheirer & Dearing [39] posit that sustained external grants are one of the two major funding streams for organizations seeking to sustain health care interventions.

Our findings suggest that health facilities relied on traditional funding partners such as PEPFAR and The Global Fund to finance core ART service delivery in-puts such as ARV drugs and commodities. The role of the Uganda government although indirect is critical as it provides base funding for items such as workforce salaries and operation costs such as utilities and infrastructure. Due to the integration of Global Fund grants into national budgeting for medicines financing in Uganda, it is plausible that their role may be more pronounced than a facility-level perspective permits. Our findings demonstrate that for components of their ART programs not funded by government or GHI partners such as the treatment of opportunistic infections and nutrition support, providers sought supplemental funding from alternative sources.

Although Uganda is one of the countries which increased domestic spending on HIV treatment between 2006 and 2011 [40], it is clear that further increases in the fiscal space for HIV treatment in Uganda’s national budget are called for as reliance on GHI funding is clearly unsustainable. Innovations in HIV financing such as the AIDS levy in countries such as Zimbabwe are instructive for Uganda and other donor-dependent countries [44, 45]. Even though Uganda proposed an AIDS Trust Fund (ATF) in 2014 to be financed through a 2% levy on beers and soft drinks, this is yet to be implemented [10]. Fulfilling the Abuja Declaration where African countries committed themselves to spending a minimum 15% of their annual budgets on the health sector could further boost domestic spending on ART service delivery.

### Alternative funding mechanisms

In the context of calls for identifying alternative funding schemes to bridge the funding gap for attaining ART scale-up targets, we found that private philanthropic organizations and individual donors were important sources of additional funding for ART programs in Uganda. The AIDS Health Care Foundation (AHF) which funds HIV treatment in 40 health facilities in South Western Uganda provides an example of the potential of private philanthropic organizations in alleviating the burden on the traditional funding sources in realizing ART scale-up targets. More research in this area is warranted. The role of private philanthropic aid in HIV funding has been noted in a previous study [46].

Private for-profit health facilities present an opportunity of tapping into new domestic financing channels through a variety of funding vehicles. The alternative funding streams elicited from PFPs include special insurance schemes for HIV patients, employer-provided insurance coverage and ‘Robin Hood’ pricing mechanisms for ART services. Scaling up employer-provided medical insurance coverage through policy imperatives could potentially increase private sector contribution to bridging the funding gap for ART service delivery in Uganda as the National Health Insurance Scheme (NHIS) takes shape. In addition, expanded private insurance coverage could potentially reduce the outpatient burden in public facilities by re-distributing some of the patient loads to for-profit health facilities. In Uganda, Kakaire, T et al. [4] have proposed that HIV patients contribute towards meeting some of the costs of their care in return for more convenient services.

The study findings illustrate the importance of organizational entrepreneurship in sustaining ART programs and highlight the dynamism required to maximize funding opportunities in the external environment. Health facilities which were able to attract multiple grants had ‘program champions’ who exhibited entrepreneurial skills in sourcing for grants and built networks that supported resource mobilization for ART program continuation [47, 48]. Organizational re-positioning for resource mobilization objectives to sustain interventions is consistently reported in the literature [27, 36, 37].

Within the framework by Shediac-Rizkallah & Bone [27], our findings suggest that Not-for-profit (PNFP) providers were inclined towards a ‘supply side’ strategy of financial sustainability by relying more on external donor funding channels to sustain ART programs. From a ‘demand side’ perspective, for-profit providers showed a greater affinity for transitioning from donor funding to more client-based funding streams. The public facilities in our sample seemed inclined towards a more hybrid approach.

### Limitations

Some of the limitations which we wish to acknowledge are specific to Phase Two of the study. Within our broader mixed-methods sequential explanatory research design, the findings from the second study phase were not intended for statistical generalization. For the six health facilities which were purposively selected, we sought to contextualize our understanding of ART program funding at the organizational level of health facilities in Uganda.

The study was liable to recall bias given that we sought to examine ART funding strategies in the participating health facilities since initial ART roll-out between 2004 and 2009. Multiple measures were taken to mitigate this limitation. Interviewees were asked to pin point specific years when funding strategies or events happened. This is reflected in some of the quotes selected for this paper. In the questionnaire, we standardize a 6-year period (2009–2014) within which respondents were asked to report the number of funding sources for ART service delivery. Additionally, we relied on multiple informants (3) per health facility to compare interviewee data in order to verify key time lines relating to ART program funding. The triangulation of data from multiple sources ameliorated the limitation of recall bias.

## Conclusion

Health facilities in Uganda diversified funding sources for ART provision beyond the traditional funding partners. Participating health facilities attracted supplemental funding from a variety of sources for components of the ART program not supported by their national and GHI funding partners. ART program funding strategies were differentiated by the type of ownership of a health facility. Private philanthropic organizations and individual donors emerged as important sources of additional funding for ART service delivery in Not-for-profit and Public health facilities. Private for-profit health facilities represent an under-explored avenue for increasing domestic financing of ART service delivery through private insurance schemes and ART-specific service fees. The alternative funding mechanisms identified could reduce dependence on GHI funding and increase local ownership of HIV programs in Uganda and other donor-dependent countries. Further research evaluating the potential contribution of the identified alternative financing mechanisms to bridge the global HIV funding gap is recommended.
